# Circular food system approaches can support current European protein intake levels while reducing land use and greenhouse gas emissions

**DOI:** 10.1038/s43016-024-00975-2

**Published:** 2024-05-28

**Authors:** Wolfram J. Simon, Renske Hijbeek, Anita Frehner, Renee Cardinaals, Elise F. Talsma, Hannah H. E. van Zanten

**Affiliations:** 1 Farming Systems Ecology Group, Department of Plant Sciences, Wageningen University and Research, Wageningen, The Netherlands; 2 Plant Production Systems Group, Department of Plant Sciences, Wageningen University and Research, Wageningen, The Netherlands; 3 Department of Food System Sciences, Research Institute of Organic Agriculture (FiBL), Frick, Switzerland; 4 Division of Human Nutrition and Health, Department of Agrotechnology and Food Sciences Group, Wageningen University and Research, Wageningen, The Netherlands

**Keywords:** Environmental impact, Agriculture, Sustainability, Nutrition, Climate and Earth system modelling

## Abstract

Protein transition and circular food system transition are two proposed strategies for supporting food system sustainability. Here we model animal-sourced protein to plant-sourced protein ratios within a European circular food system, finding that maintaining the current animal–plant protein share while redesigning the system with circular principles resulted in the largest relative reduction of 44% in land use and 70% in greenhouse gas (GHG) emissions compared with the current food system. Shifting from a 60:40 to a 40:60 ratio of animal-sourced proteins to plant-sourced proteins yielded a 60% reduction in land use and an 81% GHG emission reduction, while supporting nutritionally adequate diets. Differences between current and recommended total protein intake did not substantially impact minimal land use and GHG emissions. Micronutrient inadequacies occurred with less than 18 g animal protein per capita per day. Redesigning the food system varied depending on whether land use or GHG emissions were reduced—highlighting the need for a food system approach when designing policies to enhance human and planetary health.

## Abstract (web summary)

Almost half of land use and nearly three-quarters of greenhouse gas emissions can be reduced by adopting circularity principles and reducing the ratio of animal-sourced protein to plant-sourced protein from 60:40 to 40:60 in European diets.

## Main

In recent years, various actors within the European Union (EU) have actively pursued changes in the food system. Initiatives such as the European Green Deal aim to position the EU as a global leader in achieving climate neutrality^1^. Two possible approaches in redesigning the food system have received increased attention: protein transition and circularity in a food system.

Protein transition scenarios in the European context refer to the reduction of the share of animal proteins in human diets^2,3^. Today’s protein intake levels in the EU are around 82 g per capita per day, of which 49 g comes from animal products and 33 g from plant products^4^. In comparison, the European Food Safety Authority (EFSA) sets an average requirement (AR) intake of 46 g protein per capita per day^5^, indicating a protein overconsumption of 36 g per capita per day. Multiple studies indicated that eating less animal source proteins (ASP) positively affects health and the environment^6–11^.

Nevertheless, there remains a lack of consensus regarding how to strike an optimal balance between ASP and plant source proteins (PSP) to promote a healthy diet while mitigating environmental pressures. Some studies suggest that eating an entirely plant-based diet is the most sustainable^12,13^, while there remains uncertainty about the effects of ASP reduction in diets on protein and micronutrient adequacies^14^. Other studies introduced the concept of circularity and reported that animals can still play a crucial role and provide up to 30 g ASP per capita per day sustainably if used as waste stream recyclers^15–20,21^.

As such, numerous studies have examined the impact of plant-based diets on the environment, but none of them has integrated the protein transition (that is, shifting the ratio between animal and plant proteins towards a more plant-based diet) with circularity^6,8,14^. In this context, circularity refers to a system primarily focusing on producing human food and providing healthy diets. Organic waste is avoided whenever possible, and if it occurs, it is reused in the most efficient way possible as feed or as fertilizer^22,23^.

This study aims to model the optimal ratio between animal and plant proteins within circular a food system while minimizing land use (LU) or greenhouse gas (GHG) emissions. We address this objective by comparing two dietary approaches: one that maintains current protein intake and another that reduces protein intake to recommended levels. We use land use and greenhouse gas emissions as proxies for assessing environmental impact, and the EAT–Lancet diet and meeting nutritional requirements for all macro- and micronutrients in human diets (Supplementary Section 2) as indicators of healthy diets^24^. Food supplements were not included as an option. In our study, the term ‘transition’ is used to denote the end states or optimal outcomes of the protein transition, rather than the process itself.

Our results show that both land use (up to 60%) and GHG emissions (up to 81%) are most reduced when the current ASP:PSP ratio shifts from 60:40 to 40:60. The most substantial relative reductions in land use and GHG emissions were achieved not by changing ASP:PSP ratios, but by optimizing diets, cropping patterns, and animal husbandry and trade within a circular food system framework.

## Results

### Scenarios

We used the biophysical Circular Food System (CiFoS) optimization model to assess the optimal ASP:PSP ratio^25^. We developed 18 scenarios and assessed the ASP:PSP ratios under two different dietary approaches: one in which diets shift towards the EAT–Lancet diet^24^ while maintaining the current protein intake of 82 g protein per capita per day and a second approach representing the EAT–Lancet diet but shifting towards a recommended protein intake of 46 g protein per capita per day based on the AR from EFSA^5^. ASP:PSP ratios started at the current level, 60:40—the reference level—and were reduced in steps of 20% towards a plant-based diet while minimizing nutrient deficiencies. The 18 scenarios were compared with a reference scenario (Table 1). The reference scenario matches statistical data related to the current food system (for example, current crop production is fixed) while minimizing the difference with the current food supply^4,26,27^ as an objective function.

**Table 1:** Protein levels per protein transition scenario

Scenario names	Protein intake level	ASP:PSP ratio	PI (g per capita per day)	ASP intake (g per capita per day)
Reference_60:40	Current protein intake	60:40	82	49
LU or GHG_Cur_60:40	Current protein intake	60:40	82	49
LU or GHG_Cur_40:60	Current protein intake	40:60	82	33
LU or GHG_Cur_22:78	Current protein intake	22:78	82	18
LU or GHG_Cur_20:80	Current protein intake	20:80	82	16
LU or GHG_Cur_0:100	Current protein intake	0:100	82	0
LU or GHG_Rec_60:40	Recommended protein intake	60:40	46	28
LU or GHG_Rec_40:60	Recommended protein intake	40:60	46	18
LU or GHG_Rec_20:80	Recommended protein intake	20:80	46	9
LU or GHG_Rec_0:100	Recommended protein intake	0:100	46	0

Circularity practices were applied to all scenarios except for the reference (‘Reference_60:40’). Cur, current intake; Rec, recommended intake; PI, protein intake.

### Environmental impact of ASP:PSP ratios

**Fig. 1:**
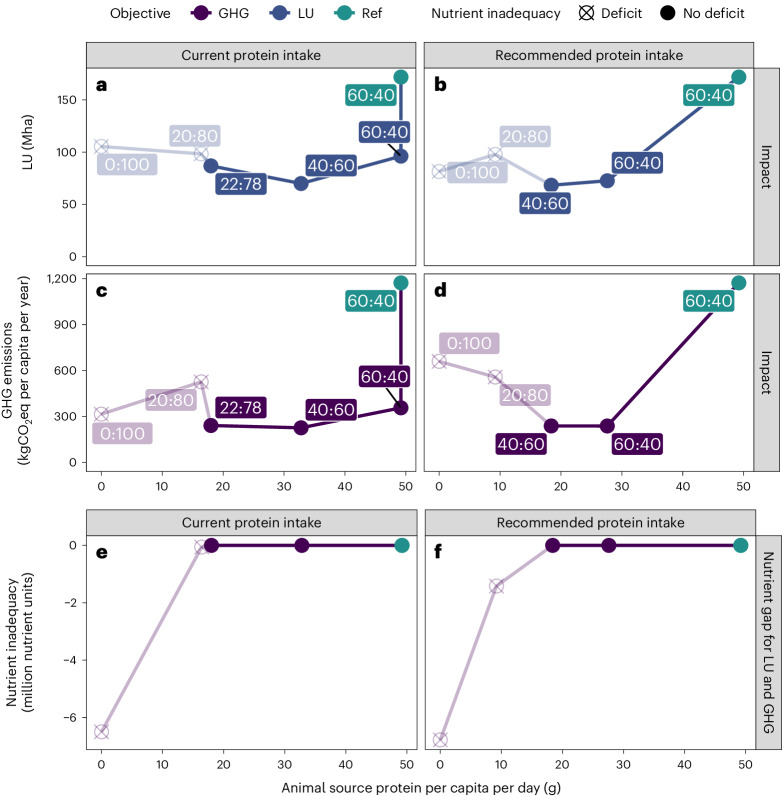
Land use and greenhouse gas emissions under different protein intake scenarios and shares of animal to plant protein in diets. **a**–**d**, Land use (**a**,**b**) and GHG emissions (**c**,**d**) along a stepwise reduction of ASP in different protein intake scenarios. **e**,**f**, A representation of the nutrient gap for each protein transition step. Ref, reference scenario. Transparent lines indicate nutrient inadequacies.

Our results show three main findings (Fig. 1a–f). First, the most considerable relative reduction in land use (44%) and GHG emissions (70%) was achieved by optimizing consumption, production and trade towards a circular food system while maintaining the current ASP:PSP ratio of 60:40 (Fig. 1a,c). Second, applying circularity principles plus shifting the ASP:PSP ratio towards more PSP reduces land use by up to 60% (Fig. 1b) and GHG emissions by up to 81% (Fig. 1c). The effect of the protein intake on land use and GHG emissions was minimal (Fig. 1a–d). Third, a plant-based diet below 18 g ASP per capita per day resulted in nutrient inadequacies with increased environmental impacts. This increase stemmed from efforts to avoid those nutrient inadequacies, leading to expanded land use and higher GHG emissions due to the cultivation of additional nutrient-dense crops (Fig. 1a–f).

#### Redesigning the food system with circularity

Redesigning the food system, including the application of circularity principles, reduced land use by 44% (from 172 Mha to 96 Mha) and GHG emissions by 70% (from 1,172 kgCO_2_eq per capita per year to 357 kgCO_2_eq per capita per year) at current protein intake levels and the current 60:40 ASP:PSP ratio (Fig. 1a,c). This reduction originates from many changes in the food system, for example, improved use of food waste streams (such as as animal feed), optimized plant and animal production systems and related decreases in transport. At the recommended protein intake, land use was reduced by 58% (172 Mha to 72 Mha), while GHG emissions decreased by 80% (1,172 kgCO_2_eq per capita per year to 238 kgCO_2_eq per capita per year) (Fig. 1b,d).

#### Optimal ASP:PSP ratio

At current protein intake levels, land use was lowest for 33 g ASP per capita per day at an ASP:PSP ratio of 40:60 with a land use of 70 Mha, which resulted in a reduction of 59% compared with the current land use of 172 Mha (Fig. 1a). The overall lowest land use, however, was achieved under the recommended protein intake approach when combining circularity with a reduced ASP:PSP ratio of 40:60 (18 g ASP per capita per day), which resulted in a 60% reduction in land use from 172 Mha to 68 Mha (Fig. 1b).

The largest reduction in GHG emissions was also achieved with an ASP:PSP ratio of 40:60 for current and recommended protein intake levels (33 g ASP per capita per day and 18 g ASP per capita per day, respectively). For current and recommended protein intake, GHG emissions were reduced similarly by 81% and 80% to 227 kgCO_2_eq per capita per year and 239 kgCO_2_eq per capita per year, respectively (Fig. 1c,d). For recommended protein intake, the emissions were the same for the ASP:PSP ratios of 40:60 and 60:40 (Fig. 1d).

#### Nutrient inadequacy in plant-based diets

**Fig. 2:**
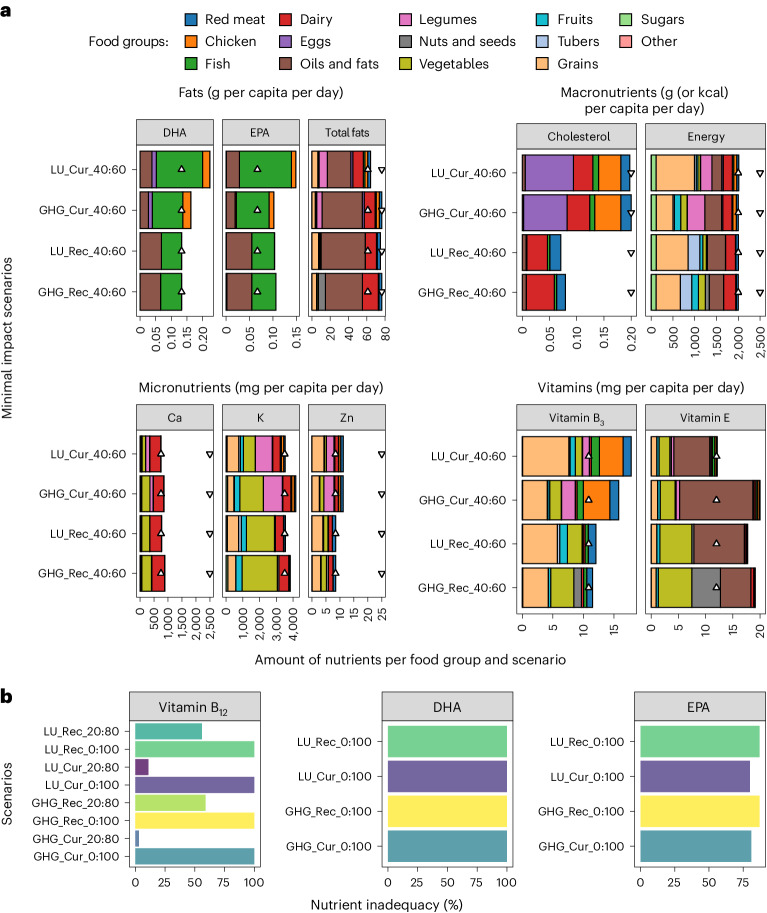
A selection of macro and micronutrients per food group sources. **a**, Different fats, energy, micronutrients and vitamins. In the graphical representation, triangles pointing upwards denote the minimum required amounts of each nutrient, representing the lower bounds, while triangles pointing downwards signify the maximum safe amounts, indicating the upper bounds. **b**, Nutrient inadequacy in percentage per inadequate nutrient and scenario (scenarios with <18 g ASP per capita per day). Colours in **b** depend on the different scenarios. EPA, eicosapentaenoic acid; DHA, docosahexaenoic acid.

Nutrient inadequacy emerged consistently below a daily intake of 18 g ASP per capita per day. Decreasing ASP further led to not only increased nutrient inadequacy but also increased land use and GHG emissions. This increase is due to a higher demand for nutrient-rich (for example, calcium, zinc, vitamin K, vitamin B_3_) crops, such as legumes, vegetables, nuts and seeds, and fruits, to compensate for the reduction of animal source food (Fig. 2). The primary nutrients leading to inadequacies were vitamin B_12_, eicosapentaenoic acid (EPA) and docosahexaenoic acid (DHA) (inadequately supplied below 18 g ASP per capita per day), and calcium, vitamin B_3_ and energy (at the border to nutrient inadequacy; Fig. 2).

### Food system redesigns at optimal ASP:PSP ratios

Transitioning the food system towards optimal ASP:PSP ratios (40:60) requires redesigning the human diet, crop production and animal production systems. When scenarios have two optimal ratios (that is, the recommended protein intake scenarios when minimizing GHG emissions), we chose to analyse the lower ratio (40:60) to understand better the effect of changing the ASP:PSP ratios on the food system.

#### Dietary strategies to reduce land use and GHG emissions

Vegetables are the only food group for which consumption increased in all optimal scenarios to shift to healthier diets due to their high contents of micronutrients and the EAT-Lancet food group requirements. However, their contribution to total protein intake is limited in all the optimal scenarios (4–10 g per capita per day). Regarding overall supply, dairy (11–13 g per capita per day) and grains (11–24 g per capita per day) are the leading protein suppliers in the optimal scenarios (Table 2).

**Table 2:** The amount of protein sourced per food group for the four optimal ASP:PSP ratios when minimizing land use and GHG emissions at different protein intake levels and with the FAO reference baseline

Food group	FAO reference (g per capita per day)	LU_Cur_40:60	LU_Rec_40:60	GHG_Cur_40:60	GHG_Rec_40:60
Red meat	13	5 (−62%)	5 (−62%)	5 (−62%)	4 (−69%)
Chicken	8	10 (25%)	0 (−100%)	11 (38%)	0 (−100%)
Fish	5	4 (−20%)	2 (−60%)	3 (−40%)	1 (−80%)
Dairy	19	11 (−42%)	12 (−37%)	11 (−42%)	13 (−32%)
Eggs	4	3 (−25%)	0 (−100%)	3 (−25%)	0 (−100%)
Oil and fat	1	0 (−100%)	0 (−100%)	0 (−100%)	0 (−100%)
Legumes	4	20 (400%)	1 (−75%)	28 (600%)	0 (−100%)
Nuts and seeds	2	0 (−100%)	0 (−100%)	0 (−100%)	3 (50%)
Vegetables	3	4 (33%)	8 (167%)	9 (200%)	10 (233%)
Fruits	1	0 (−100%)	1 (0%)	1 (0%)	1 (0%)
Tubers	2	0 (−100%)	1 (−50%)	0 (−100%)	0 (−100%)
Grains	20	24 (20%)	16 (−20%)	11 (−45%)	13 (−35%)
Sugars	0	0 (0%)	0 (0%)	0 (0%)	0 (0%)

The percentage shows the relative increase (+) or decrease (−) when comparing the optimal scenarios to the FAO reference scenario.

At current protein intake levels, the reduction in land use and GHG emissions is mainly due to a shift in protein sources. Compared with the FAO reference intake, red meat and egg consumption was reduced by 62% and 25%, respectively, when land use and GHG emissions were minimized. Dairy protein was reduced by 43% when land use and GHG emissions were minimized. In comparison, fish was reduced by 20% when minimizing land use and 40% when minimizing GHG emissions. Only chicken meat protein increased by 25% and 38% for minimizing land use and GHG emissions. For plant proteins, legumes and vegetables were the only increasing protein sources. Legumes in diets increased by 400% and 600% when land use and GHG emissions were minimized. We found an increase of 33% and 200% for vegetables when minimizing land use and GHG emissions, respectively. Grains are an essential protein source when minimizing land use (reduced by 20%) but less when minimizing GHG emissions (reduced by 45%; Table 2).

At recommended protein intake levels, ASP was reduced to 18 g per capita per day (ASP:PSP ratio of 40:60) when land use and GHG emissions were minimized compared with 49 g today. Dairy (12–13 g per capita per day) and grains (13–16 g per capita per day) were again the primary protein sources, followed by vegetables and red meat (Table 2). Lowering the protein intake to recommended levels increased the risk of nutrient inadequacies. At an ASP:PSP ratio of 40:60, calcium, vitamin B_12_ and zinc are going towards nutrient inadequacy, driving the model to increase food sources with higher amounts of these scarce nutrients (Fig. 2).

#### Cropping strategies for reducing land use and GHG emissions

**Fig. 3:**
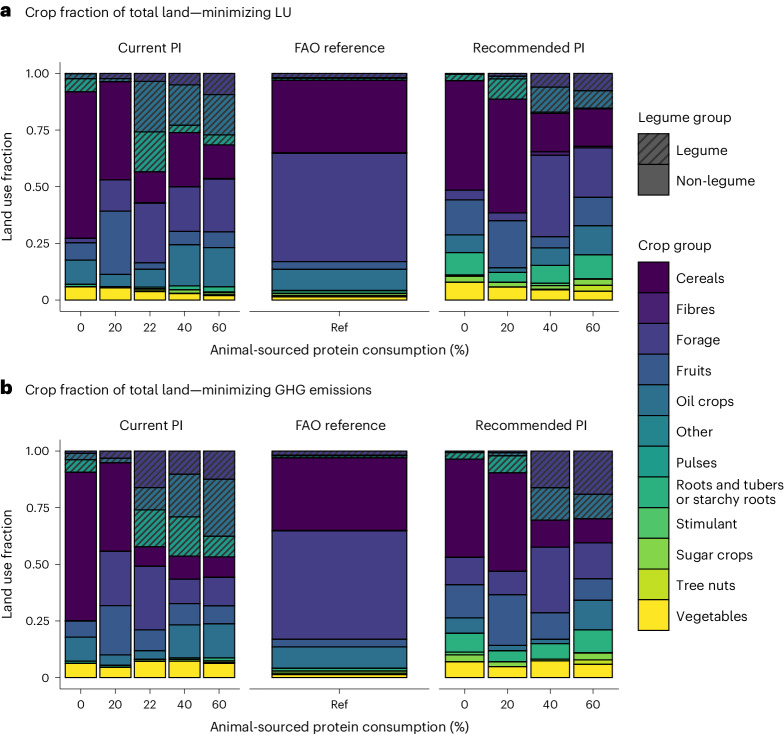
Relative crop shares of agricultural land per crop group, protein intake level and environmental impact category. **a**,**b**, Relative share of crop groups of total agricultural land when minimizing LU (**a**) and minimizing GHG emissions (**b**). Grassland was considered in the crop group ‘Forage’. Ref, FAO reference scenario.

**Extended Data Fig. 1:**
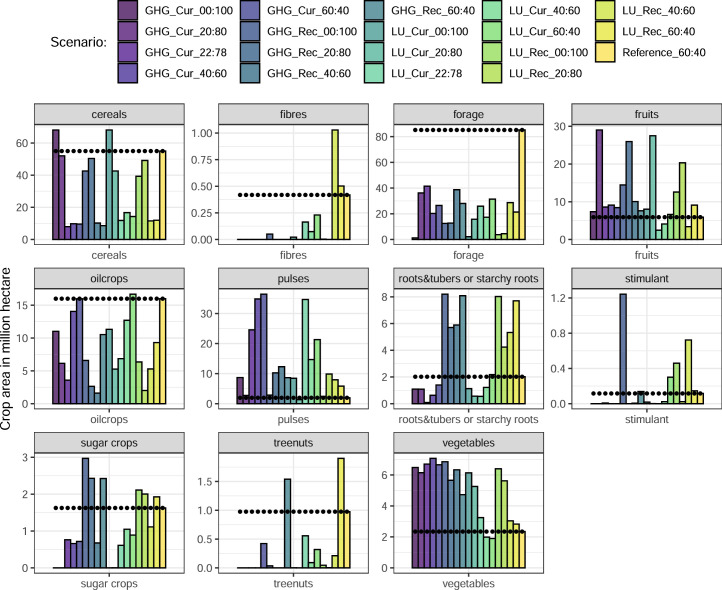
Croping area per scenario and crop group. Dots indicate the reference baseline.

One key factor in reducing land use and GHG emissions in the optimal scenarios was increasing legume production, especially soybeans. The increase in legumes is due to their high protein content (up to 36 g protein per 100 g for soybeans), their favourable amino acid profile (especially for soybeans) and the ability of legume crops to fix atmospheric N, thereby reducing the amount of artificial fertilizer required and associated GHG emissions. At current protein intake levels, the relative land share of legumes to all other crops increased by a factor of 6 (to 30 Mha of the 96 Mha) to cover one-third of the arable land when reducing land use and a factor of 9 (to 45 Mha of the 96 Mha) to cover half of the arable land when reducing GHG emissions (Fig. 3 and Extended Data Fig. 1). Although at the recommended protein intake level, cereals were favoured over legumes as a protein source, the production of legumes still increased 2 times (to 11 Mha of the 72 Mha) when reducing land use and 5 times (to 26 Mha of the 86 Mha) when reducing GHG emissions. Furthermore, vegetable and oil crops increased considerably under both dietary approaches in transitioning towards healthier diets. At the same time, forage crops and permanent grassland decreased in land share. Grassland decreased strongly from 82 Mha to 14 Mha and 25 Mha at the current and recommended protein intake, respectively.

#### Rearing strategies for reducing land use and GHG emissions

**Extended Data Fig. 2:**
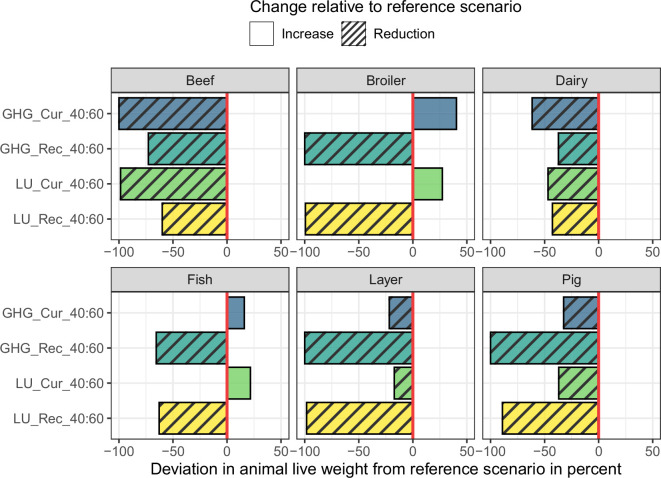
Relative deviation in crude protein to reference in percentage and per animal type for the optimal scenarios. Relative deviation in live weight from reference scenario in percentage and per animal types Beef, Broiler, Dairy, Fish, Layer, and Pig. The red line represents the reference (0% deviation). The stripes indicate a relative reduction of the animal type per scenario compared to the reference scenario.

Overall, animal numbers were predominately reduced. However, at current protein intake levels, farmed fish and broilers were slightly increased. Layers were only slightly reduced and thus are land-efficient animals within a circular food system. Layers, broilers and farmed fish have the most synergy between low land use and GHG emissions. Dairy production decreased by approximately 50% across all optimal scenarios. However, it remained a staple in the food system, offering a wide range of nutritious products (for example, milk, yogurt, butter, meat, offal) through the upcycling of human-inedible biomass such as grass within a circular system (Extended Data Fig. 2).

At recommended protein intake at the 40:60 ASP:PSP ratio, diets are constrained to 18 g ASP per capita per day. This low amount of ASP eliminates pigs, layers and broilers from the food system while minimizing land use and GHG emissions. Only fish, dairy and beef remained in the system at around half of the initial baseline animal numbers (Extended Data Fig. 2).

#### Transportation strategies to reduce emissions

**Extended Data Fig. 3:**
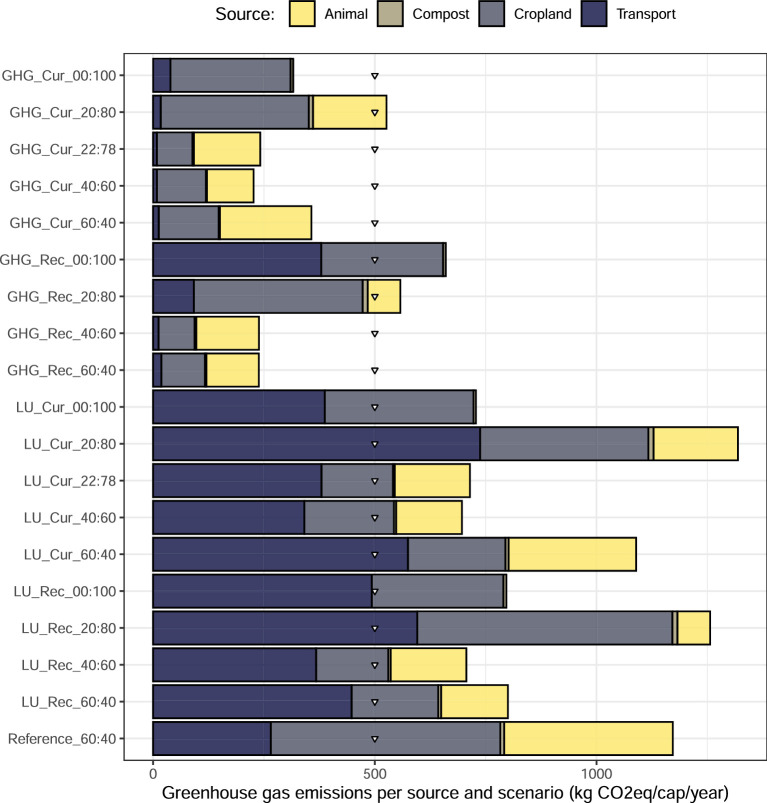
Greenhouse gas emissions (GHG) by scenario and source. Dots indicate the sustainable amount of emissions from the food system per person and year (500 kgCO2eq/capita/year).

Our results show that a highly effective strategy to reduce GHG emissions is cutting down on transportation. In the optimal GHG-minimizing scenarios, the share of transportation to the total GHG emissions was less than 5%, compared with around 50% in the reference scenario. However, transitioning towards a more plant-based diet in the food system increases transportation emissions. The acquisition of location-specific and nutrient-rich crops exclusively cultivated in certain areas of the EU28 necessitates sourcing food items from more distant regions (Extended Data Fig. 3).

## Discussion

### The potential of applying circularity principles

Although an ASP:PSP ratio of 40:60 results in the lowest land use and GHG emissions, our results show that the most considerable relative reduction can be achieved when redesigning the food system with circular principles. Both land use and GHG emissions can be reduced by 44% and GHG emissions by 70% without changing the total protein intake or share of ASP. This shift towards a more circular food system leads to changes in plant and animal production systems and related reductions in transport. For instance, while the overall consumption of ASP remains constant, its composition changes; chicken and fish intake in diets rises, whereas red meat, dairy and egg intake declines. Organic waste is avoided whenever possible, and if it occurs, it is efficiently reused as feed or fertilizer. Our findings support recent studies showing that agricultural land can largely be spared when feeding farmed animals primarily with low-cost biomass such as by-products, food waste and crop residues^22^. This spared land could be used for other purposes, such as sequestering carbon through reforestation, which could also increase biodiversity^28^.

### The optimal ASP:PSP ratio of 40:60

The optimal ratio for reducing land use and GHG emissions was 40:60, regardless of the protein intake level. This shift reduced land use and GHG emissions by 60% and 81% at the current and recommended protein intake. Protein intake levels did not have a substantial environmental impact as the difference between the minimal impact scenarios of current and recommended protein intake for land use and GHG emissions amounted to only 2 Mha and 12 kgCO_2_eq per capita per year. When looking at the literature, we can find similar approximations of ideal ASP:PSP ratios ranging from 20:70 to 50:50 (refs. ^14,29^).

### Eliminating nutritional inadequacy in plant-based diets

This study shows that, when shifting towards plant-based diets, we must balance micronutrients and macronutrients to ensure nutritionally adequate diets. In diets with less than 18 g ASP per capita per day, nutrient inadequacies are more likely in a suite of (predominantly animal source) micronutrients such as vitamin B_12_, calcium, EPA and DHA. The finding of nutrient inadequacies when going more plant based is supported by several studies, although the mentioned ranges of adequate ASP:PSP ratios differ considerably from 50:50 (refs. ^14,30^) to 15:85 (ref. ^29^). The latter aligns with our boundary of 22:78 (18 g ASP per capita per day) at the current protein intake, in which we identified the boundary to nutrient inadequacy. The shortage of nutrients drives GHG emissions and land use, which is supported in the literature^31,32^. Adding supplementation, fortification and future foods to our suite of dietary options can reduce impacts on land use and GHG emissions when transitioning towards more plant-based diets, yet these were not included in this study (Supplementary Discussion 1).

### Strategies for cutting land use and greenhouse gas emissions

**Extended Data Fig. 4:**
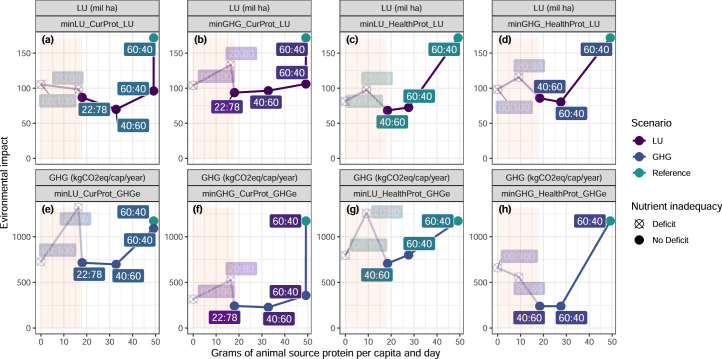
Protein transition with land use when minimising GHG emissions and GHG emissions when minimising land use for current and recommended protein intake. (a) Land use when minimising land use at current protein intake; (b) Land use when minimising GHG at current protein intake; (c) Land use when minimising land use at recommended protein intake; (d) Land use when minimising GHG at recommended protein intake; (e) Greenhouse gas emissions when minimising land use at current protein intake; (f) Greenhouse gas emissions when minimising GHG at current protein intake; (g) Greenhouse gas emissions when minimising land use at recommended protein intake; (h) Greenhouse gas emissions when minimising GHG at recommended protein intake. Red shadings represent nutrient inadequacies.

Food system redesigns are complex, and, as our results reveal, both trade-offs and synergies occur between minimizing land use and GHG emissions (Extended Data Fig. 4). Strategies to reduce both land use and GHG emissions were as follows: (1) reduce the amount of ASP in diets by 33% at the current protein intake and by 42% at the recommended protein intake to an ASP:PSP ratio of 40:60, (2) reduce animal numbers drastically and (3) prioritize the cultivation of legumes and vegetables. An example of a trade-off is the cultivation of legumes when they exceed 40% of agricultural land in reducing GHG emissions. Legumes require more land for the same amount of protein as cereals owing to lower yields but decrease GHG emissions owing to less fertilizer needed. This trade-off could be reduced by increasing legume competitiveness with novel breeding strategies. Our results also reveal a trade-off related to transportation as land use increases with local production owing to decreased yields. Transitioning towards more sustainable energy sources could overcome this trade-off (Supplementary Discussion 3).

### ASP:PSP ratio in national food-based dietary guidelines

Changing ASP:PSP ratios will have considerable environmental and human nutritional benefits in combination with a balanced diet. National food-based dietary guidelines (FBDG) often do not consider environmental sustainability in their recommendations^33^; however, this is changing. Although most European FBDG recommendations advise eating less red and processed red meat and replacing it with legumes, white meat and fish^34^, recommendations regarding overall protein intake levels are still high. The Netherlands, for example, advises a protein intake of 98 g per capita per day (45 g ASP per capita day) in its FBDG, and Sweden, 85 g per capita per day (56 g ASP per capita per day)^35^. These high ASP levels clearly show the potential for further reducing the ASP:PSP ratios in FBDGs in Europe to reduce land use and GHG emissions of the food system. Our results show that it would be most beneficial to reduce the national recommendations of ASP to at least 33 g per capita per day and to stimulate a change in the type of ASP source consumed.

## Outlook

Our study has shown the potential of incorporating circularity principles with a protein transition, emphasizing the necessity of adopting a comprehensive food system approach. While policymakers might set the 40:60 ratio as a target, the multifaceted nature of its implications, as highlighted in our results, suggests that its implementation can lead to diverse outcomes. Sheer advocacy for the 40:60 ratio without comprehensive guidance could result in unintended trade-offs, potentially undermining policymakers’ objectives related to land use, GHG emission reductions and other impacts. It is crucial to underscore the significance of an integrated food system approach to foster synergies and mitigate potential trade-offs from excessive focus on singular environmental impacts. This study has shown that food system modelling offers insights into the interactions between circularity and protein transition and can help inform the sustainable redesign of food systems.

## Methods

### Circular food system model

This study is based on the CiFoS model^22^. CiFoS is a biophysical food system optimization model coded in the General Algebraic Modeling System (GAMS) that incorporates circular principles owing to its unique model structure^25^. In CiFoS, human-inedible by-products can be used as a fertilizer or feed for farmed animals, and this model facilitates the choice of food system redesign by minimizing a selected environmental impact (in this study, land use or GHG emissions) or minimizing the difference to current protein food supplies while fulfilling all nutrient requirements in human diets. In our protein scenarios, the model selects food items from plants, farmed animals and captured fish that lead to minimal land use or GHG emissions. The CiFoS model contains various scales, from the EU28 or a country to an agroecological zone. The CiFoS model consists of several modules: human system, crop system and farmed animal system, including aquaculture, captured fisheries, residual streams, transportation and GHG emissions from different sources. These are described in more detail in the following sections.

#### Human nutrition

In CiFoS, the daily nutrient requirements recommended by the EFSA for the EU28 are met to ensure a nutritious diet. The model covers 37 nutritional indicators, including macro- and micronutrients, vitamins, amino acids, fatty acids and energy content (Supplementary Section 2). Vitamin D and iodine recommendations were excluded as a nutritional requirement owing to mandatory salt fortification for iodine in the EU and implicit limitations in obtaining enough vitamin D from diets alone. The nutritional content of the CiFoS products is based on the FoodData Central Data from United States Department of Agriculture (USDA)^36^. In addition to nutrient requirements, food intake constraints per product and food family were included based on the reference range of the EAT–Lancet diet^24^. Apart from the reference scenarios, all scenarios complied with the EFSA nutrient requirements and the EAT–Lancet diet^24^. The model includes 155 different human-edible products that can be produced and used to create healthy and sustainable diets from various animal and plant sources. The many food items allow CiFoS to design diets that match the objective function (primarily to reduce environmental impacts).

#### Land availability and land use change

The total agricultural land in the EU28 is 172 Mha (ref. ^27^). CiFoS distinguishes three land types: arable, marginal grassland and rangelands. Arable land can also be cultivated with temporary grassland. This grassland is treated like any other crop in the crop rotation. To define arable land, we used the cropland map from the International Institute for Applied Systems Analysis (IIASA) and International Food Policy Research Institute (IFPRI)^37^, while for pasture land and rangelands, we used the land cover maps from the History Database of the Global Environment^37,38^. Grassland was classified as temporary when cropland and grassland land cover maps overlapped. Land use change from deforestation for gaining croplands or pasture is not allowed in the CiFoS model. Grass on arable land is treated like a crop and can be changed to cropland. Permanent grassland and rangeland cannot be altered to arable land. Crop and grassland cannot be expanded into other land use types such as natural areas or forests. Expanding crops or grasslands can, therefore, never lead to deforestation.

#### Cropping system

CiFoS includes 43 food crops and 8 fodder crops, including 3 different grass types (temporary, permanent and rangeland). Yield and harvested area data of the 43 food crops are based on the Global Spatially-Disaggregated Crop Production Statistics Data for 2010 (Version 2.0), further referred to as Spatial Production Allocation Model (SPAM)^39,40^. Yield and harvested area data for the fodder, tree nuts and vegetable (red, green and other) crops were sourced from the EARTHSTAT dataset ‘Harvested Area and Yield for 175 Crops’^41^. Both of these crop production data sets represent current yield intensity levels. Yields and area data were spatially extracted for agroecological climate–soil zones. These zones were created based on the intersection of the global agroecological zones^42^ and the Intergovernmental Panel on Climate Change (IPCC) default soil classes derived from the Harmonized World Soil Data Base^43^. In CiFoS, all crops can be freely selected on a climate–soil-zone level.

#### Crop rotations

In this study, we assume crop rotations for annual crops. As CiFoS is not a dynamic model, we assign the number of years to each crop that needs to be between two cultivation events to not run into soil-borne diseases. Using this rotation break number, we can assign a specific maximum land share per zone for each crop when simulating the share of a crop in a rotation. We translate time into space, meaning that when a crop needs 3 years between cropping events, we allow this crop only one-third of the area. The frequency of the cultivation of the same crop in time was taken from a previous study^44^.

#### Crop fertilization

Crop fertilization considers nitrogen (N) and phosphorus (P). Fertilizer requirements per crop are calculated by the total amount of above-ground nutrients and losses. For P, we assume a fixed loss fraction of 12.5% of the fertilizer applied^45^. For N, we calculate the losses based on the IPCC, which include direct and indirect N_2_O and runoff and leaching losses^46^. These N losses are climate–soil type dependent and are calculated on a climate–soil zone level using the disaggregated emission factors from IPCC. N fixation is directly subtracted from the amount of N in the crops.

#### Fertilizer types

As this is a circularity model, we aimed to include various fertilizers to represent all the side-waste streams in a food system. CiFoS includes the following fertilizers that can be used to meet the crop nutrient requirements on a climate–soil zone level: by-products from processing, manure, compost, human excreta, above-ground crop residues and artificial fertilizer. Aside from these fertilizers, N deposition and N fixation are also included in our modelling framework. N deposition is a fixed N input extracted per climate–soil zone, while biological N fixation is calculated using the following formula following an adjusted approach from ref. ^47^:

$$\begin{array}{c}{{\mathrm{N}}\;{\mathrm{fixation}}}\left({\mathrm{kg}}\;/{\mathrm{ha}}^{-1}\right)={\mathrm{Ndfa}}/100\times {\mathrm{Y}}/{\mathrm{NHI}}\end{array}$$

where Ndfa is the percentage of N uptake derived from biological N fixation, *Y* is the harvested yield (expressed in kgN ha^−1^ yr^−1^) and NHI is the N harvest index, defined as the ratio of the harvested material to the total above-ground production.

In the baseline, we fix the amount of N and P from artificial fertilizers based on the International Fertilizer Association^48^. The amount of sludge available for agriculture is derived from Eurostat^49^. The nutrient content in the manure is calculated as a function of feed intake and the nutrients retained as milk, meat and eggs. For compost, we calculated the nutrient content similarly, taking the nutrient content of the organic composting inputs (that is, food waste) and subtracting the N losses (‘Food losses and waste’). The N and P contents of crop residues are based on the USDA nutrient tool database.

#### Animal system

Animal production systems include dairy, beef, pigs, broilers and farmed fish (fresh and salt water)^50^. We used Nile tilapia and Atlantic salmon production data as proxies for fresh and salt water species. Livestock systems include three different intensity levels, while farmed fish represent the current productivity levels. For each animal type and productivity level, we use adjusted animal nutrient requirements. When producing an animal, the model also calculates the animal nutrient requirements of the whole herd structure, such as reproductive stock (that is, heifers in a dairy system) and parent stock (that is, sows in a pig system). Different feed ingredients can be selected to meet those requirements, such as co-products, food waste, grass resources, animal by-products and, if not otherwise used in the food system, high-quality biomass such as grains, which humans can consume. The feed ratio is a model outcome. The animal source food output from the animal production systems depends on three aspects: the quantity and quality of biomass and grass resources available for farm animals, the ability of animals to convert biomass streams into animal source food and the nutrient requirements of humans.

#### Captured fisheries

Captured fisheries can be chosen as an additional product of animal source food for human food and animal feed (only by-products). Maximum landings of captured fisheries are derived from the dataset Official Nominal Catches 2006–2021 provided by the International Council for the Exploration of the Sea (ICES)^51^. We distinguished between edible yield fractions of all landed fish and their non-human-edible by-products to avoid feed–food competition.

#### Residual streams

##### Crop residues

Next to the harvested yield, crops also produce crop residues, the fraction of the above-ground residues that are not harvested as the main product^46^. In our study, crop residues can be applied only as fertilizer locally in the same climate–soil zone in which the crop was produced.

##### Processing by-products

Plant and animal source food products are processed into the main product (that is, wheat flour) and by-products (that is, wheat bran). These processing fractions are mainly derived from the technical conversion factor document of FAO^52^. One crop can have multiple potential main products (that is, white wheat flour, whole-grain wheat flour, wheat grains) with each set of by-products. Animal by-products are a fraction of the live weight output of each farmed animal system^50^. By-products can be used as animal feed or fertilizer.

##### Food losses and waste

Waste losses are calculated along all supply chain stages, including post-harvest, processing and packaging, distribution and retail, and consumption losses^53^. Post-harvest, processing and packaging, and distribution of waste occur in the country of production. Food losses and waste are allowed to be fed only to monogastric animals and fish to minimize food safety hazards.

##### Manure

All farmed animals (except farmed fish) are assumed to produce manure. The nutrient content of manure is a function of feed intake and retained nutrients in the live animal. All manure, except the one excreted while grazing, is considered to enter a manure management system (MMS). Manure from the MMS can be used throughout the country. Grazing ruminants fertilize the land they are grazing on with their droppings.

##### Human excreta

Currently, 36% of sludge produced in EU28 is applied on agricultural land^49^. We keep this share constant throughout all scenarios. We assumed nutrient contents for sludge of 7.5% and 1.2% of N and P, respectively^54^.

#### Transportation

Food, feed and their associated by-products can be moved between EU28 countries via lorries. By contrast, food waste, manure and grassland are mandated to be used within the country where they are produced. The underlying assumption was that crop and livestock products would undergo processing into food, feed and by-products within the country of origin before being transported to the destination country for consumption. The distances between countries are calculated based on the centroids of each member state and the distances separating them.

#### GHG emissions

##### GHG emissions from the animal production system

We used IPCC tier 2 methodologies to compute GHG emissions^55^. GHG emissions stemming from farmed terrestrial animals (dairy, beef, pigs, broilers and layers) encompassed both methane (CH_4_) and nitrous oxide (N_2_O) emissions originating from the management of livestock manure. Livestock manure management results in considerable amounts of CH_4_ and N_2_O emissions. To calculate methane emissions from manure management, we used a formula that involves multiplying the volatile solid excretion by the methane conversion factor (specific to each MMS), B0 (representing the maximum methane production capacity of manure) and 0.67 (to convert methane from cubic metres to kilograms of CH_4_). Volatile solid excretion was determined by considering the digestibility of protein and organic matter present in the feed consumed by each animal species. The CiFoS model endogenously calculates the amount of animal feed consumed. As for N_2_O, emissions from manure encompass both direct and indirect emissions, with the latter arising from the volatilization of ammonia and N_2_O. The calculation of N excretion involved subtracting the N retained in meat, milk or eggs from the total N intake. To determine N_2_O emissions, we then multiplied the N excretion by the appropriate emission factor, which varies depending on the species and the type of housing system in use. The N_2_O emissions are considered emissions generated within the aquaculture system for farmed fish. Unconsumed feed and excreta containing N (calculated as the difference between N intake and N retained in body tissues) were multiplied by 1.8% and converted from N to N_2_O (refs. ^43,56^). In addition, in the case of ruminant systems, we considered CH_4_ emissions from enteric fermentation and N_2_O emissions resulting from grassland fertilization. The calculation for methane emissions from enteric fermentation involved multiplying the gross energy intake by Ym (representing the proportion of gross energy in feed converted to CH_4_) and dividing by 55.65 (which signifies the gross energy content of methane). This approach aligns with the IPCC tier 2 methodology^43^. Nitrous oxide emissions originating from grassland encompassed both direct and indirect emissions. The latter arises from the volatilization of ammonia and N_2_O, as well as the leaching of nitrate. These emissions stem from N fertilization and manure release during grazing^46^.

##### GHG emissions from cropping systems

The cultivation of crops causes the release of N_2_O and CO_2_ emissions. We used the IPCC tier 1 methodology to calculate crop emissions^46^. Regarding N fertilization of crops, the type of fertilizer, soil characteristics and climate conditions all play a role in determining the levels of N_2_O emissions. N_2_O emissions can be direct and indirect, with indirect emissions arising from the volatilization of ammonia and N_2_O and nitrate leaching into the environment. To calculate N_2_O emissions, we multiplied the fertilizer amount applied by the corresponding emission factor. This emission factor varies based on the specific type of N fertilizer used and the climate–soil zone.

In addition, we accounted for emissions associated with the production of synthetic fertilizers using data from the ecoinvent database^57^. In the emission calculations, we also considered N_2_O emissions from drained organic soils, considering factors such as land use, climate zone and soil type (whether peat or non-peat soil)^43,46,58^. It is important to note that we did not include CH_4_ emissions from rice cultivation, as they were considered negligible in our analysis. Furthermore, we did not factor in CO_2_ emissions from crop management (that is, fuel for tractors).

##### GHG emissions from compost

The process of composting food waste leads to the release of N_2_O and CH_4_ emissions. To estimate N_2_O emissions, we used the N content in the food waste and the N loss fraction of 38% and converted the amount of N lost into N_2_O. CH_4_ emissions were derived from the N losses by converting the N content in the compost into carbon based on the average compost’s carbon-to-nitrogen (C:N) ratio, which is ideally around 15. Subsequently, we converted the carbon content into the total CH_4_ emitted^59^.

##### GHG emissions from transportation

The transportation of crops, fish, food, by-products, manure and food waste involves burning fossil fuels, which releases CO_2_ emissions. We measured the distances between countries, specifically from one country’s centre point to another’s centre point, and quantified the total ton-kilometres involved in this transportation. The ton-kilometres were multiplied by an emission factor derived from the ecoinvent database^57^. To calculate the total GHG emissions, we aggregated them into CO_2_eq, considering a 100 year time horizon (GWP100). For CH_4_, a factor of 28 and, for N_2_O, a factor of 265, were used. The results provided GHG emission totals for EU28 as a whole and per capita per year^46^.

### Circular principles

Circularity presents a systemic solution by reducing unavoidable waste streams such as food waste and overconsumption of nutrients. If waste is unavoidable, waste streams are reused in the most sustainable manner possible. In addition, food processing produces by-products such as wheat middlings during flour production. These by-products and waste streams can be used as compost to reduce the need for artificial fertilizers. Furthermore, if waste streams are used as feed for farmed animals, inedible biomass for humans could be transformed into livestock products and manure, leading to increased ecosystem services due to improved soil fertility and less pressure on land^60,61^. The fundamental principles of circular food systems are centred around avoiding and reusing waste and by-product streams to close biomass and nutrient cycles.

In this study, circularity was modelled as follows: farmed animals can be fed by organic side streams^17^. Second, to avoid food waste, the edible ratio of animals is increased, meaning that humans consume all edible parts of the farmed animals (that is, offal), and overconsumption is avoided. Third, nutrient recycling is improved by fostering circular fertilization, such as using leguminous crops in crop rotation compost from organic waste streams and crop residues to reduce artificial fertilizer inputs.

### Scenario description

#### Reference scenario

The reference scenario fixes the current agricultural land from FAOSTAT and minimizes the difference in the FAO protein supply per food group^4^. The reference scenario uses net import (import–export) data of food and feed products from the Food Balance Sheet (FBS) as a fixed inflow to the food system^4^. Trade was allowed only between countries but not outside the study boundaries. The reference is, therefore, a self-sufficient production and consumption scenario for the EU28 countries. The agricultural land is based on the MAPSPAM data^39^ and was scaled to the total FAOSTAT areas per land use^26,27^. The current protein supply was derived from the FBS element: ‘protein supply quantity (g per capita per day)’^4^.

#### Protein intake scenarios

**Extended Data Fig. 5:**
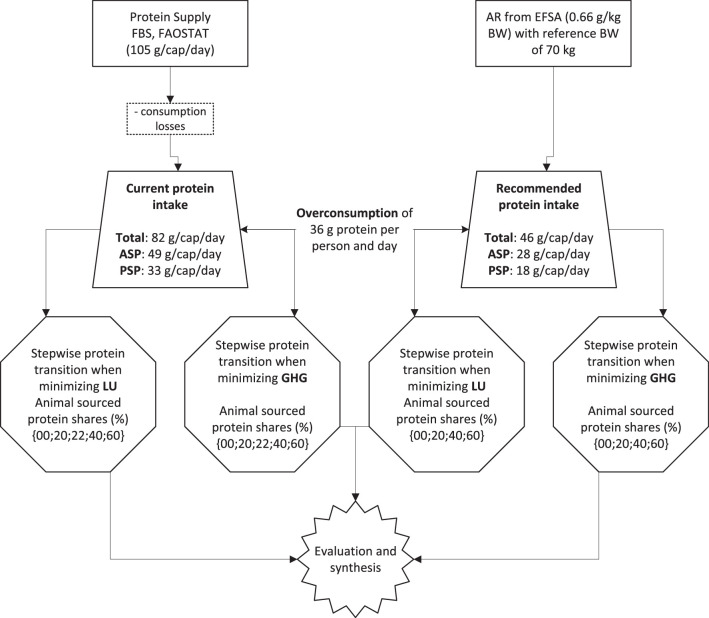
Modelling workflow and main data source for defining current and recommended protein intake. Abbreviations: EFSA = European Food Safety Authority, FBS = Food Balance Sheet, PRI = Population reference intake, BW = Body weight, LU = Land use, GHG = Greenhouse gas emissions, ASP = Animal source protein, PSP = Plant source protein.

Two protein intake levels were defined to assess the effect of these levels on land use and GHG emissions at a food system level. Current protein intake was calculated by subtracting consumption losses from the protein food supply based on food groups (FAO-FBS element: ‘protein supply quantity (g per capita per day)’)^4,53^. The current EU28 protein intake resulted in 82 g protein per capita per day. Recommended protein intake was calculated using the EFSA AR of 0.66 g protein per kg body weight with a 70 kg reference body weight^5^. This calculation resulted in a recommended protein intake of 46 g protein per capita per day; thus, the difference between current and recommended protein intake is 36 g protein per capita per day. The current ratio of around 60:40 between animal and plant source proteins was calculated from the FBS^4^. To derive the amount of animal protein intake, current and recommended protein intake levels were multiplied by 0.6, resulting in an ASP intake of 49 g per capita per day and 28 g per capita per day, respectively (Extended Data Fig. 5).

#### Protein transition scenarios

The protein transition from the current to a plant-based food system is modelled as a stepwise reduction of ASP in the diet. The transition is modelled in four steps, going from an ASP:PSP ratio of 60:40, 40:60, 20:80 to 0:100% (plant based). An additional ASP:PSP ratio of 22:78 was added for current protein intake levels because the 20:80 ratio was nutrient inadequate (<18 g ASP per capita per day), which led to difficulties compared with the other ratios.

### Objective functions and final scenario definitions

We expanded the initial food system model developed by ref. ^22^ to quantify the effect of the protein transition in EU28 on land use and GHG emissions. The adjusted model included a double optimization option: first, minimizing the human nutrition gap, then minimizing land use or GHG emissions. In this manner, we ensured that model outcomes closely met the nutritional requirements for macro- and micronutrients (Extended Data Fig. 5 and equations in Supplementary Section 1). To model all the scenarios, we use four different objective functions combined in three optimization scenario options (see ‘Objective function equations’ in Supplementary Section 1):

1. Minimizing the positive and negative deviation to the FBS protein supply while fixing the current total agricultural land
2. Minimizing the human nutrient gap and then minimizing land use
3. Minimizing the human nutrient gap and then minimizing GHG emissions

Using one reference scenario, two different optimization approaches, two protein intake scenario levels, four protein transition step scenarios and the two ‘22:78’ scenarios, we generated a total of 19 scenarios. The basic assumptions of the scenarios are shown in Extended Data Table 1.

### Software and data analysis

All data transformation, analysis and visualization were performed using R (version 4.2.2)^62^. The optimization modelling was performed using the GAMS^25^.

### Reporting summary

Further information on research design is available in the Nature Portfolio Reporting Summary linked to this article.

### Supplementary information

Supplementary Information Supplementary Sections 1 and 2, Discussions 1–3 and references.

Reporting Summary

## Figures and Tables

**Extended Data Table 1:**
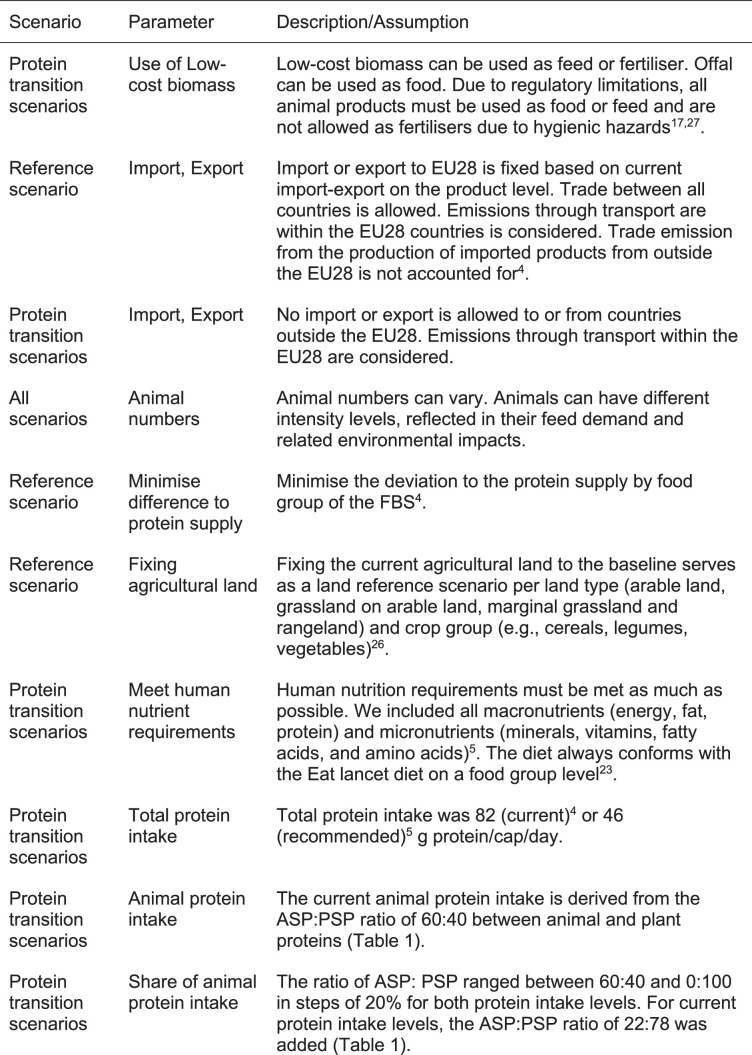
Scenario parameters and associated assumptions for the modelling procedure

Abbreviations: ASP = Animal source protein, PSP = Plant source protein, FBS = Food Balance Sheet. References refer to the reference section in the main article.

## Data Availability

The raw data are available from the corresponding author on request. Datasets used in this paper are as follows: FoodData Central Data from USDA (https://fdc.nal.usda.gov/download-datasets.html); Food Supply: FAO FBS (https://www.fao.org/faostat/en/#data/FBS); EAT–Lancet diet ranges per food group (https://eatforum.org/content/uploads/2019/07/EAT-Lancet_Commission_Summary_Report.pdf); FAO Crops and Livestock Products (QCL) (https://www.fao.org/faostat/en/#data/QCL); land use cover map IIASA-IFPRI cropland map (https://geo-wiki.org/Application/index.php); grassland cover maps, History Database of the Global Environment 3.3 (https://geo.public.data.uu.nl/vault-hyde/HYDE%203.3[1710493486]); SPAM, Global Spatially-Disaggregated Crop Production Statistics Data for 2010 Version 2.0 (https://dataverse.harvard.edu/dataset.xhtml?persistentId=doi:10.7910/DVN/PRFF8V); Harvested Area and Yield for 175 Crops Year 2000 (https://s3.us-east-2.amazonaws.com/earthstatdata/HarvestedAreaYield175Crops_Geotiff.zip); Agro-ecological Zones, 33-classes, GAEZ (v.4) (https://s3.eu-west-1.amazonaws.com/data.gaezdev.aws.fao.org/LR.zip); IPCC Default Soil Classes Derived from the Harmonized World Soil Data Base, version 1.2 (https://data.isric.org/geonetwork/srv/api/records/41cb0ae9-1604-4807-96e6-0dc8c94c5d22); Global and Regional Phosphorus Budgets in Agricultural Systems and Their Implications for Phosphorus-Use Efficiency, PANGAEA (10.1594/PANGAEA.875296); N_2_O Emissions from Managed Soils, and CO_2_ Emissions from Lime and Urea Application (https://www.ipcc-nggip.iges.or.jp/public/2019rf/pdf/4_Volume4/19R_V4_Ch11_Soils_N2O_CO2.pdf); N and P Consumption of Artificial Fertilizers Is Based on the IFA (https://www.ifastat.org/databases/plant-nutrition); Sewage Sludge Production and Disposal, Eurostat (https://data.europa.eu/data/datasets/g1a4auwbnkfrmzm3dg6zg?locale=en); Captured fisheries data from the ICES Database on Official Nominal Catches 2006-2021, Version 10-07-2023 (https://ices.dk/data/dataset-collections/Pages/Fish-catch-and-stock-assessment.aspx); processing fractions: Technical Conversion Factor document of FAO (https://www.fao.org/3/cb2466t/cb2466t.pdf); losses at all supply chain stages, including post-harvest, processing and packaging, distribution and retail, and consumption losses (https://www.fao.org/3/i2697e/i2697e.pdf); synthetic fertilizer production: ecoinvent database (https://ecoquery.ecoinvent.org/3.10/cutoff/search); manure management emissions for livestock (https://www.ipcc-nggip.iges.or.jp/public/2019rf/pdf/4_Volume4/19R_V4_Ch10_Livestock.pdf); compost emissions (10.1177/0734242x09345275); transportation emissions: ecoinvent database (https://ecoquery.ecoinvent.org/3.10/cutoff/search); and livestock data (10.1016/j.jclepro.2019.01.329).

## Code availability

The model code is available from the corresponding author on request.
