# Supplementary material for: Circular food system approaches can support current European protein intake levels while reducing land use and greenhouse gas emissions
Source: Nat Food. 2024 May 28;5(5):402–12. doi: 10.1038/s43016-024-00975-2 (PMC11132985; doi:10.1038/s43016-024-00975-2)
Supplement: Supplementary file 1 — Supplementary Sections 1 and 2, Discussions 1–3 and references. [file 43016_2024_975_MOESM1_ESM.pdf]

nature food

Supplementary information https://doi.org/10.1038/s43016-024-00975-2

Circular food system approaches can support current European protein intake levels while reducing land use and greenhouse gas emissions

In the format provided by the authors and unedited

Supplementary Information

Supplementary information

SI1. Objective function equations

Minimise positive and negative deviation from current agricultural land

$$DeviationToProteinSupply = \sum_{C} \sum_{FG} \begin{bmatrix} PositiveDeviationToProteinSupply_{C,FG} \\ +NegativeDeviationToProteinSupply_{C,FG} \end{bmatrix}$$

where C is Country, FG is Food Group.

Minimise human nutrient gap

$$TotalHumanNutrientGap = \sum_{C} \sum_{N} \left(HumanFoodNutrientGap_{C,N}\right) / MinimumNutrientRequirement_{N}$$

where C is Country and N is human food macro and micronutrients used in the model.

Minimise Land use

$$TotalLand = \sum_{C} \sum_{Z} \sum_{S} \sum_{L} \sum_{Crop} \left(AgriculturalLand_{C,Z,S,L,Crop}\right)$$

where C is Country, Z is Climate Zones, S is Soil type, L is agricultural land type and Crop is the all the crops used in the model.

Minimise GHG emissions

$$TotalGHGEmissions = \sum_{C} \left(GHGEmissionsPerCountry_{C}\right)$$

where C is Country.

SI2. Nutritional indicators used to generate nutritionally adequate diets in the CiFoS model

Fiber, Energy, Protein, Carbohydrates, Fat, Linoleic Acid (LA), Alpha-Linolenic Acid (ALA), Docosahexaenoic Acid (DHA), Cholesterol, Sodium (Na), Calcium (Ca), Magnesium (Mg), Iron (Fe), Copper (Cu), Selenium (Se), Zinc (Zn), Vitamin A, Vitamin B1 (Thiamine), Vitamin B2 (Riboflavin), Vitamin B3 (Niacin), Vitamin B6, Vitamin B9 (Folate), Vitamin B12, Vitamin E, Vitamin K, Nitrogen (N), Phosphorus (P), Potassium (K), Histidine (HIS), Isoleucine (ILE), Leucine (LEU), Lysine (LYS), Methionine (MET), Phenylalanine (PHE), Threonine (THR), Tryptophan (TRP), Valine (VAL).

Supplementary Discussion

SD1: Nutrient inadequacies in plant-based diets

A review study complements our findings further, stating that the risk for nutrient inadequacies for vitamin B12, calcium, zinc and selenium in plant-based diets is high[7]. It is relevant to understand whether these nutrient inadequacies can be mitigated with fortified food products or future foods like seaweeds, insects and cultured meat to substitute animal source micronutrients in plant-based diets[8–11] while providing an environmentally friendly solution[12,13]. Other studies report that future food diets in the EU can reduce land use (-87%) and GHG emissions (-83%) compared to current production systems[12,14,13]. Thus, food-based strategies like fortification, biofortification and dietary diversification can improve micronutrient intake. Thus, adding food fortification and future foods to our suite of dietary options can reduce impacts on land use and GHG emissions when transitioning towards more plant-based diets, yet these were not included in this study[15,16].

SD2: Sensitive model assumptions and future outlook

In the context of GHG assessments, we faced the challenge of dealing with various evaluation metrics. Given our objective to evaluate long-term impacts, we have chosen the GWP100 metric, primarily due to its focus on long-term environmental impacts[17]. Although GWP* may offer a more accurate depiction of the effects of short-lived climate pollutants like methane, it is less appropriate for evaluating long-term environmental outlooks[18].

Another assumption of this study is that although requirements for individual amino acids were included, we did not directly consider protein quality and digestibility. These aspects may affect the results as the fraction of total protein taken up by the body will be lower in plant-based diets[19,20]. This simplification may imply that the protein recommendation would be higher than presented in this study. Nevertheless, the EFSA protein recommendations are based on a mixed EU diet and already assume a particular share of protein from plant sources[5]. Careful planning is needed to ensure that both macronutrients and micronutrients remain adequate. From that perspective, a protein intake above the average requirement can help avoid nutrient inadequacies if the share of PSP in EU28 diets is increased.

SD3: Strategies for cutting land use and greenhouse gas emissions

Trade-offs and synergies with other environmental impacts or ecosystem services will also exist. For example, reducing animal proteins in diets might increase water demand, as a recent systematic review suggested[21]. Besides these environmental impacts, there may also be trade-offs with economic and social factors that are beyond the scope of this study.

References

1. Bakaloudi, D. R. *et al.* Intake and adequacy of the vegan diet. A systematic review of the evidence. *Clinical Nutrition* **40**, 3503–3521 (2021).

2. Bak, U. G. Seaweed cultivation in the faroe islands: An investigation of the biochemical composition of selected macroalgal species, optimised seeding technics, and open-ocean cultivation methods from a commercial perspective. (2019).

3. Croft, M. T., Lawrence, A. D., Raux-Deery, E., Warren, M. J. & Smith, A. G. Algae acquire vitamin B12 through a symbiotic relationship with bacteria. *Nature* **438**, 90–93 (2005).

4. Kanazawa, A. VITAMINS IN ALGAE. *NIPPON SUISAN GAKKAISHI* **29**, 713–731 (1963).

5. Mišurcová, L., Ambrožová, J. & Samek, D. Chapter 27 - seaweed lipids as nutraceuticals. in *Advances in food and nutrition research* (ed. Kim, S.-K.) vol. 64 339–355 (Academic Press, 2011).

6. Tzachor, A. Novel foods for human and planetary health. *Nature Food* **3**, 247–248 (2022).

7. Parodi, A. *et al.* The potential of future foods for sustainable and healthy diets. *Nature Sustainability* **1**, 782–789 (2018).

8. Mazac, R. *et al.* Incorporation of novel foods in european diets can reduce global warming potential, water use and land use by over 80. *Nature Food* **3**, 286–293 (2022).

9. Eustachio Colombo, P., Elinder, L. S., Lindroos, A. K. & Parlesak, A. Designing nutritionally adequate and climate-friendly diets for omnivorous, pescatarian, vegetarian and vegan adolescents in sweden using linear optimization. *Nutrients* **13**, (2021).

10. Gazan, R. *et al.* Individual diet optimization in french adults shows that plant-based ‘dairy-like’ products may complement dairy in sustainable diets. *Sustainability* **14**, 2817 (2022).

11. Manzano, P. *et al.* Challenges for the balanced attribution of livestock’s environmental impacts: The art of conveying simple messages around complex realities. *Animal Frontiers* **13**, 35–44 (2023).

12. Cain, M. *et al.* Improved calculation of warming-equivalent emissions for short-lived climate pollutants. *npj Climate and Atmospheric Science* **2**, 29 (2019).

13. Adhikari, S., Schop, M., De Boer, I. J. M. & Huppertz, T. Protein quality in perspective: A review of protein quality metrics and their applications. *Nutrients* **14**, 947 (2022).

14. Barré, T. *et al.* Integrating nutrient bioavailability and co-production links when identifying sustainable diets: How low should we reduce meat consumption? *PLOS ONE* **13**, e0191767 (2018).

15. EFSA Panel on Dietetic Products, N. & Allergies. Scientific opinion on dietary reference values for protein. *EFSA Journal* **10**, 2557 (2012).

16. Jarmul, S. *et al.* Climate change mitigation through dietary change: A systematic review of empirical and modelling studies on the environmental footprints and health effects of 'sustainable diets'. *Environ Res Lett* **15**, 123014 (2020).
