# Supplementary material for: Circular food system approaches can support current European protein intake levels while reducing land use and greenhouse gas emissions
Source: Nat Food. 2024 May 28;5(5):402–12. doi: 10.1038/s43016-024-00975-2 (PMC11132985; doi:10.1038/s43016-024-00975-2)
Supplement: Supplementary file 2 — Reporting Summary [file 43016_2024_975_MOESM2_ESM.pdf]

nature portfolio

Corresponding author(s): Wolfram Simon

Last updated by author(s): Mar 31, 2024

Reporting Summary

Nature Portfolio wishes to improve the reproducibility of the work that we publish. This form provides structure for consistency and transparency in reporting. For further information on Nature Portfolio policies, see our Editorial Policies and the Editorial Policy Checklist.

Statistics

For all statistical analyses, confirm that the following items are present in the figure legend, table legend, main text, or Methods section.

n/a	Confirmed	
☒	☐	The exact sample size (*n*) for each experimental group/condition, given as a discrete number and unit of measurement
☒	☐	A statement on whether measurements were taken from distinct samples or whether the same sample was measured repeatedly
☒	☐	The statistical test(s) used AND whether they are one- or two-sided<br>*Only common tests should be described solely by name; describe more complex techniques in the Methods section.*
☒	☐	A description of all covariates tested
☒	☐	A description of any assumptions or corrections, such as tests of normality and adjustment for multiple comparisons
☒	☐	A full description of the statistical parameters including central tendency (e.g. means) or other basic estimates (e.g. regression coefficient) AND variation (e.g. standard deviation) or associated estimates of uncertainty (e.g. confidence intervals)
☒	☐	For null hypothesis testing, the test statistic (e.g. F, t, r) with confidence intervals, effect sizes, degrees of freedom and P value noted<br>*Give P values as exact values whenever suitable.*
☒	☐	For Bayesian analysis, information on the choice of priors and Markov chain Monte Carlo settings
☒	☐	For hierarchical and complex designs, identification of the appropriate level for tests and full reporting of outcomes
☒	☐	Estimates of effect sizes (e.g. Cohen's d, Pearson's r), indicating how they were calculated

Our web collection on statistics for biologists contains articles on many of the points above.

Software and code

Policy information about availability of computer code

Data collection	No software was used.
Data analysis	Data was prepared, analyzed and visualized using the free open source software R: A Language and Environment for Statistical Computing (version R-4.2.2, https://cran.r-project.org/bin/windows/base/). Optimization modeling was done using the GAMS GAMS Release 46.3.0 (https://www.gams.com/).

For manuscripts utilizing custom algorithms or software that are central to the research but not yet described in published literature, software must be made available to editors and reviewers. We strongly encourage code deposition in a community repository (e.g. GitHub). See the Nature Portfolio guidelines for submitting code & software for further information.

Data

Policy information about availability of data

All manuscripts must include a data availability statement. This statement should provide the following information, where applicable:

- Accession codes, unique identifiers, or web links for publicly available datasets
- A description of any restrictions on data availability
- For clinical datasets or third party data, please ensure that the statement adheres to our policy

The raw data have been deposited in a GIT repository and are available on request under a license similar to Creative Commons Attribution-Non Commercial-Share A like 4.0 International Public License.

Datasets used in this paper were: FoodData Central Data from USDA (Access: https://fdc.nal.usda.gov/download-datasets.html); Food supply: FAO Food Balance Sheet (FBS) (Access: https://www.fao.org/faostat/en/#data/FBS); EAT-Lancet diet ranges per food group (Access: https://eatforum.org/content/uploads/2019/07/EAT-Lancet_Commission_Summary_Report.pdf); FAO Crops and livestock products (QCL) (Access: https://www.fao.org/faostat/en/#data/QCL); Land use cover map 'IIASA-IFPRI cropland map (Access: https://geo-wiki.org/Application/index.php); Grassland cover maps, History Database of the Global Environment 3.3 (Access: https://geo.public.data.uu.nl/vault-hyde/HYDE%203.3[1710493486]); Spatial Production Allocation Model (SPAM), Global Spatially-Disaggregated Crop Production Statistics Data for 2010 Version 2.0 (Access: https://dataverse.harvard.edu/dataset.xhtml?persistentId=doi:10.7910/DVN/PRFF8V); Harvested Area and Yield for 175 Crops year 2000 (Access: https://s3.us-east-2.amazonaws.com/earthstatdata/HarvestedAreaYield175Crops_Geotiff.zip); Agro-ecological zones, 33-classes, GAEZ (v.4) (Access: https://s3.eu-west-1.amazonaws.com/data.gaezdev.aws.fao.org/LR.zip); IPCC default soil classes derived from the Harmonized World Soil Data Base, version 1.2 (Access: https://data.isric.org/geonetwork/srv/api/records/41cb0ae9-1604-4807-96e6-0dc8c94c5d22); Global and regional phosphorus budgets in agricultural systems and their implications for phosphorus-use efficiency. PANGAEA (Access: https://doi.org/10.1594/PANGAEA.875296); N2O emissions from managed soils, and CO2 emissions from lime and urea application (Access: https://www.ipcc-nggip.iges.or.jp/public/2019rf/pdf/4_Volume4/19R_V4_Ch11_Soils_N2O_CO2.pdf); N and P consumption of artificial fertilisers based is based on the IFA (Access: https://www.ifastat.org/databases/plant-nutrition); Sewage sludge production and disposal, EUROSTAT (Access: https://data.europa.eu/data/datasets/g1a4auwbnkfrmzm3dg6zg?locale=en); RAM Legacy Stock Assessment (Access: https://www.re3data.org/repository/r3d100012095) and FAO marine capture data (Access: https://www.fao.org/fishery/en/topic/166235). Processing fractions: FAO's technical conversion factor document (Access: https://www.fao.org/3/cb2466t/cb2466t.pdf); Losses at all supply chain stages, including post-harvest, processing and packaging, distribution and retail, and consumption losses (Access: https://www.fao.org/3/i2697e/i2697e.pdf); Synthetic fertiliser production: ecoinvent database (Access: https://ecoquery.ecoinvent.org/3.10/cutoff/search); Manure management emissions for livestock (Access: https://www.ipcc-nggip.iges.or.jp/public/2019rf/pdf/4_Volume4/19R_V4_Ch10_Livestock.pdf); Compost emissions (Access: https://doi.org/10.1177/0734242x09345275); Transportation emissions: eco-invent database (Access: https://ecoquery.ecoinvent.org/3.10/cutoff/search); Livestock data (Access: https://doi.org/10.1016/j.jclepro.2019.01.329).

Human research participants

Policy information about studies involving human research participants and Sex and Gender in Research.

Reporting on sex and gender	N/A
Population characteristics	N/A
Recruitment	N/A
Ethics oversight	N/A

Note that full information on the approval of the study protocol must also be provided in the manuscript.

Field-specific reporting

Please select the one below that is the best fit for your research. If you are not sure, read the appropriate sections before making your selection.

☐ Life sciences ☐ Behavioural & social sciences ☒ Ecological, evolutionary & environmental sciences

For a reference copy of the document with all sections, see nature.com/documents/nr-reporting-summary-flat.pdf

Ecological, evolutionary & environmental sciences study design

All studies must disclose on these points even when the disclosure is negative.

Study description

The objective of this research is to determine the most favorable proportion of animal and plant proteins in human diets, taking into account two different levels of protein consumption. The study was conducted on a comprehensive scale, encompassing all EU28 countries (EU27+UK). The sustainability of the food system was evaluated by examining two key measures: land use and greenhouse gas emissions. This study is based on the Circular Food Systems model (CiFoS). CiFoS is a bio-physical data- driven food system linear programming optimization model coded in GAMS. The model was developed to represent a circular food system with all its subsystems such as human nutrition, animal and plant-production, capture and fisheries, and waste streams.

Research sample

Nutrition data: In CiFoS, the daily recommended nutrient requirements advised by the European Food Safety Agency (EFSA) for the EU28 are met to ensure a nutritious diet. The model covers 37 nutritional indicators including macro and micronutrients, vitamins, amino acids, fatty acids and energy content. Vitamin D and iodine recommendations were excluded as a nutritional requirement due to mandatory salt fortification for iodine in the EU and implicit limitations in obtaining enough vitamin D from diets alone. Nutritional content of the CiFoS products is based on the FoodData Central Data from USDA. In addition to nutrient requirements, food intake constraints per product and/or food family were included based on the reference range of the EAT-Lancet dietary guidelines.

Land availability: Land cover maps for grassland were taken from the History Database of the Global Environment (HYDE) and represent the year 2010, while the cropland was taken from IIASA-IFPRI (https://doi.org/10.1111/gcb.12838).

Plant production: CiFoS includes 43 food crops and 8 fodder crops including 3 different grass types. Production data of the 43 food crops are based on the Global Spatially-Disaggregated Crop Production Statistics Data for 2010 (Version 2.0) further referred to as SPAM. Production data for the fodder crops were sourced from the EARTHSTAT dataset "Harvested Area and Yield for 175 Crops". Yields and area data were spatially extracted for climate-soil zones. These zones were created based on the intersection of the Global

Agro-ecological Zones and the IPCC default soil classes derived from the Harmonized World Soil Data Base. Fertilization is assumed to be balanced, meaning that we only fertilize as much as the nutrient uptake of the plants plus the losses. The losses for nitrogen were calculated based on the IPCC.

Animal production: The animal system includes livestock (dairy, beef, pigs, broilers and layers) and farmed fish (Atlantic salmon and Nile tilapia) on the basis of https://doi.org/10.1016/j.jclepro.2019.01.329. Nutritional values of the animal feed are sourced from CVB database (https://vvdb.cvbdiervoeding.nl/Manage/Tools/VwCalc.aspx).

Fisheries: The model further includes capture fisheries as food and feed. Capture fisheries provide fish for human consumption and fish by-products which can be fed to animals. Landings of capture fisheries are based on a combined database of the RAM Legacy Stock Assessmentand FAO marine capture data.

Greenhouse gas emissions: Greenhouse gas emissions arise from cropping and animal production systems and transportation. GHG emissions from cropping systems are based on the direct and indirect emissions from N2O in relation to soil and climate and fertilizer type59. For animal GHG emissions, we used the tier2 approach from the IPCC methodology. The transportation of crops, animals and by-products is allowed between EU28 countries. These GHG emissions are the result of transportation fossil fuel use. The emissions were calculated using the distance between countries, the lorry size, and the emission factor per kilometre from evo-invent. All emissions were converted to CO2 equivalents and summed to calculate the total amount of GHG emission per food system.

Reference scenario: The reference scenario fixes the current agricultural land from FAOSTAT and minimizes the difference to the FAO protein supply per food group. Trade was only allowed between countries but not outside the study boundaries. The reference is therefore a self-sufficient production and consumption scenario for the EU28 countries. The agricultural land is based on the MAPSPAM data and was scaled to the total FAOSTAT areas per land use. The current protein supply was derived from Food Balance Sheet (FBS) element: “protein supply quantity (g/cap/day)”).

Sampling strategy	*Note the sampling procedure. Describe the statistical methods that were used to predetermine sample size OR if no sample-size calculation was performed, describe how sample sizes were chosen and provide a rationale for why these sample sizes are sufficient.*
Data collection	We used exclusively existing data from online sources. No field work to collect data was performed.
Timing and spatial scale	We model a timeframe of one year.<br>For crop production data we used a multiple year range around 2010 as this was the latest dataset we could find with the parameters needed (MapSPAM, Earthstat). For all other datasets we collected the latest data available (around 2020-2022).
Data exclusions	No data was excluded.
Reproducibility	All data, data processing and modeling was documented and version controlled on a gitlab repository. All data preparation, analysis and visualization was done in R which allows to revisit all parts of the code at any time. The whole manuscript is written in R markdown which allows to revisit the underlying calculation of any number in the manuscript. GAMS is also a programming language which again allows to follow each line of the model code used. All these measures allow to reproduce the whole modeling and reporting procedure performed to produce the submitted paper.
Randomization	This is a food system modeling study which did not use any statistical methods to anlayse the data. Randomisation is therefore not applicable.
Blinding	We did not do any experiments so blinding was not applied.

Did the study involve field work? ☐ Yes ☒ No

Reporting for specific materials, systems and methods

We require information from authors about some types of materials, experimental systems and methods used in many studies. Here, indicate whether each material, system or method listed is relevant to your study. If you are not sure if a list item applies to your research, read the appropriate section before selecting a response.

Materials & experimental systems

n/a	Involved in the study	
☒	☐	Antibodies
☒	☐	Eukaryotic cell lines
☒	☐	Palaeontology and archaeology
☒	☐	Animals and other organisms
☒	☐	Clinical data
☒	☐	Dual use research of concern

Methods

n/a	Involved in the study	
☒	☐	ChIP-seq
☒	☐	Flow cytometry
☒	☐	MRI-based neuroimaging
